# Inflammatory Arthritis Induced by Pembrolizumab in a Patient With Head and Neck Squamous Cell Carcinoma

**DOI:** 10.3389/fonc.2018.00409

**Published:** 2018-09-26

**Authors:** Nikolaos Spathas, Panagiota Economopoulou, Myrto Cheila, Ioannis Kotsantis, Antonis Fanouriakis, Dimitra Kassara, Amanda Psyrri

**Affiliations:** ^1^Section of Medical Oncology, Department of Internal Medicine, Faculty of Medicine, National and Kapodistrian University of Athens, Attikon University Hospital, Athens, Greece; ^2^4th Department of Internal Medicine, Rheumatology and Clinical Immunology, Faculty of Medicine, National and Kapodistrian University of Athens, Attikon University Hospital, Athens, Greece

**Keywords:** pembrolizumab, inflammatory arthritis, head and neck cancer, immune checkpoint inhibitors, immune-related adverse events

## Abstract

**Introduction:** T cell checkpoint inhibitors targeting Programmed cell Death protein-1 (PD-1) have emerged as novel immunotherapy agents showing remarkable efficacy in head and neck squamous cell carcinoma (HNSCC). Despite important clinical benefits, they are associated with side effects that occur as a consequence of general immunological stimulation due to loss of T cell inhibition. Herein, we report the unusual case of inflammatory arthritis induced by anti-PD-1 agent pembrolizumab.

**Case report:** A 55-years old male was treated with pembrolizumab at a dose of 200 mg every 3 weeks for a metastatic hypopharyngeal carcinoma. Following two cycles of immunotherapy, and while complete response of lung metastases was achieved, the patient presented with stiffness, swelling and pain of the right knee. Clinical examination and synovial fluid analysis revealed a seronegative inflammatory arthritis. Pembrolizumab therapy was interrupted and low-dose prednisone was administered with remarkable clinical improvement. Pembrolizumab was reintroduced, but after the fifth cycle, the patient developed inflammatory polyarthritis involving both knees and interphalangeal joints of both hands resulting in severe clinical deterioration. At that time, treatment with pembrolizumab was permanently discontinued. High-dose prednisone and methotrexate treatment led to remission of clinical symptoms.

**Conclusion:** Pembrolizumab-induced inflammatory arthritis is an unusual rheumatic immune-related adverse event that physicians are likely to encounter as ICI use expands. Multidisciplinary management and rheumatology consultation are necessary to provide immediate treatment and avoid permanent joint damage.

## Introduction

Immunotherapy has shown to provide durable responses for patients with advanced cancer (1). HNSCC serves as a paradigm of immunosuppressive disease, as it is characterized by dysregulated cytokine profile, impaired function of immune effector cells, and abnormalities in tumor-associated antigen (TAA) presentation (2). In November 2016, the Food and Drug Administration (FDA) approved nivolumab, an anti-programmed cell death protein-1 (anti-PD-1) monoclonal antibody for the treatment of platinum-refractory recurrent and/or metastatic HNSCC based on a pivotal phase III clinical trial which demonstrated improved overall survival (OS) compared with chemotherapy (3). On the other hand, the anti-PD-1 pembrolizumab has failed to improve OS in a phase III trial in the same setting (4).

Despite important clinical benefits, immunotherapeutic agents are associated with a wide spectrum of side effects termed immune-related adverse events (irAEs) that occur as a consequence of general immunological stimulation due to loss of T cell inhibition (5). Among irAEs, rheumatic and myoskeletal irAEs have to date not been widely characterized. Herein, we describe a case of inflammatory polyarthritis induced by pembrolizumab in a patient with metastatic HNSCC.

## Case presentation

A 55-years-old Caucasian male patient was diagnosed with a stage IVB head and neck squamous cell carcinoma (HNSCC) in May 2015. He was a heavy smoker and social drinker with no other significant medical history, and was initially treated with concurrent cisplatin-based chemoradiotherapy.

On routine follow-up visit in September 2016, Computed Tomography (CT) scans showed lung metastases. The patient was enrolled in a clinical trial and was randomized to pembrolizumab monotherapy every 3 weeks. Following the first two cycles of immunotherapy, the patient presented with stiffness, swelling and pain of the right knee. Physical examination showed inflammatory monoarthritis, with diffuse swelling and tenderness of the right knee. Laboratory tests were remarkable for an elevated erythrocyte sedimentation rate (ESR, 40 mm/h) and C-reactive protein (CRP, 50 mg/L); rheumatoid factor (RF) and anti-cyclic citrullinated peptide (anti-CCP) antibodies were negative and serum uric acid was normal. Following rheumatologic consultation, knee joint aspiration was performed, and synovial fluid (SF) analysis revealed a yellow, cloudy appearance, decreased viscosity and a cell count of 7040 cells/mm3 with 80% neutrophils, indicating an inflammatory arthritis. SF cultures were sterile and no crystals were found on microscopy. The patient was initially treated with prednisone 5 mg twice a day with significant improvement over the following days. Inflammatory arthritis was attributed to pembrolizumab therapy and the third cycle was eventually postponed. Importantly, restaging imaging at that timepoint showed complete response of the disease.

**Figure 1:**
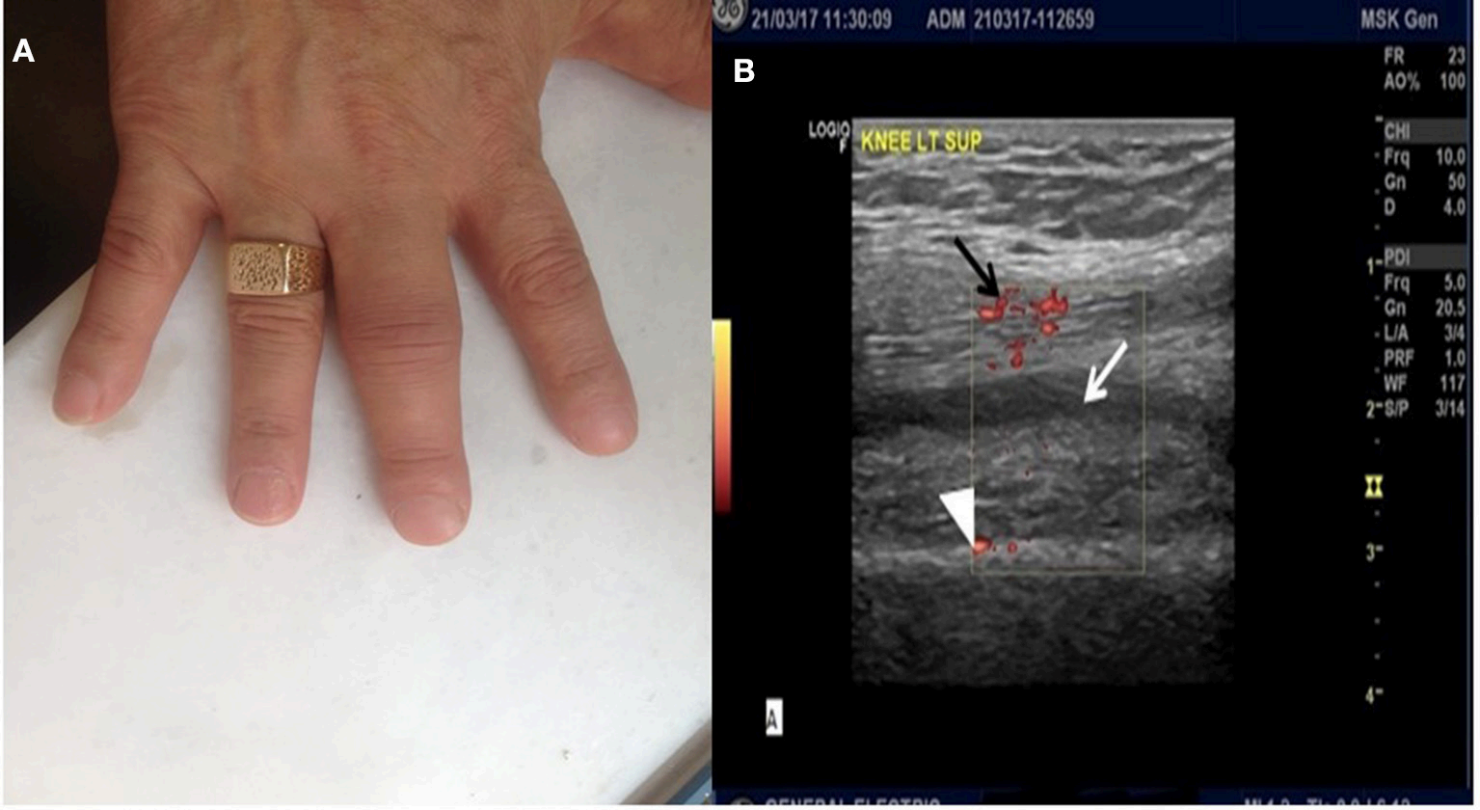
**(A)** Arthritis of interphalangeal joints of the hands. **(B)** Ultrasound image of the left knee. Note the presence of synovial fluid (*white arrow*), marked synovial hypertrophy (*white arrowhead*) and presence of Power Doppler signal (*black arrow*), all indicative of active synovial inflammation.

Following reinstitution of pembrolizumab therapy, bilateral arthritis of the knees, accompanied by arthritis of interphalangeal joints of both hands (Figure 1A), developed after the fifth cycle, Ultrasound of the knees showed evidence of active synovitis (Figure 1B) and a diagnosis of inflammatory polyarthritis was established. The patient was retreated with prednisone 5 mg twice a day and pembrolizumab therapy was interrupted. Due to the patient's clinical deterioration, and because protocol limitations did not allow increase of prednisone dose or administration of immunomodulatory drugs, pembrolizumab was permanently discontinued. Of note, the patient remained in complete remission. Methotrexate 7.5 mg po as a single weekly dose was added to control synovial inflammation and, following also pembrolizumab discontinuation, the patient's symptoms gradually improved.

A written informed consent was obtained from the patient for the publication of this case report.

## Discussion

Immune-related adverse events (irAEs) induced by cancer immunotherapy have been described in a variety of clinical settings. Rheumatic and musculoskeletal events have been less often reported in clinical trials and most cases originate from case reports or case series (6–12). Our patient experienced severe symptoms, which severely compromised his quality of life. Interestingly, pembrolizumab retreatment triggered his symptoms.

In a recent report, Capelli and colleagues reviewed published literature on rheumatic and musculoskeletal irAEs. Arthritis was reported in 5/33 clinical trials, and vasculitis was reported in only 2. Case reports include the occurrence of inflammatory arthritis, vasculitis, myositis, and lupus nephritis (7). Of 13 patients with rheumatologic iRAEs following treatment with ipilimumab, or nivolumab in another series, 9 patients developed inflammatory polyarthritis (9). Consistent with our report, RF and anti-CCP antibodies were negative, albeit three cases were positive for ANA. Two more cases of delayed onset inflammatory polyarthritis after treatment with pembrolizumab for metastatic melanoma have been reported (12).

In a recent systematic review and meta-analysis of studies performed on cancer patients receiving anti-PD-1 and anti-PD-L1 agents, only single cases of arthritis were reported in two studies at a rate of below 1% in each. However, across control groups, musculoskeletal complaints were very common, ranging from 9 to 18% for arthralgia, 2 to 16% for back pain, 4 to 6% for musculoskeletal pain, and 4 to 16% for myalgia (13). In another retrospective study that included 496 patients with metastatic melanoma who were treated with anti-PD-1 agents, arthritis was rarely reported (14, 15).

The etiology of pembrolizumab-induced inflammatory arthritis is not yet clarified. It has been suggested that anti-PD1 antibodies enhance autoimmunity by activating T cell function and may allow previously dormant arthritogenic clones to expand or newly presented autoantigens to develop into arthritogenic clones (12). Indeed, there might be a correlation between enhanced autoimmunity and response to immunotherapy treatment. Vitiligo has been correlated with increased immune response in patients receiving pembrolizumab (16). This is consistent with our case, which developed severe arthritis, but also complete disease remission.

Our case is unique in that the patient experienced severe symptoms that remarkably affected his quality of life; of note, his cancer did not cause him any symptoms. Furthermore, complete remission was achieved and remained after pembrolizumab discontinuation. We believe that severity of inflammatory arthritis correlated with increased immune response.

At present, our knowledge about the incidence and the diagnosis of rheumatic irAEs is limited. Furthermore, no consensus exists regarding the treatment of arthritis in this setting. High dose glucocorticoids might alter the efficacy of immunotherapeutic agents and immunomodulatory drugs might trigger tumor progression. Frequently, discontinuation of immunotherapy remains the only option. Although optimal duration of immunotherapy treatment has not been established in cancer, it is uncertain whether the efficacy of these drugs persists after their discontinuation. Collaboration between rheumatologists and oncologists is pivotal in order to understand the spectrum of rheumatic irAEs, optimize their treatment and define the appropriate time point of treatment discontinuation, if necessary.

## Author contributions

All authors listed have made a substantial, direct and intellectual contribution to the work, and approved it for publication.

### Conflict of interest statement

The authors declare that the research was conducted in the absence of any commercial or financial relationships that could be construed as a potential conflict of interest.

## References

[1] Finn OJ. Immuno-oncology: understanding the function and dysfunction of the immune system in cancer. Ann Oncol. (2012) 23 (Suppl. 8):viii6–9. 10.1093/annonc/mds256 22918931 PMC4085883

[2] Ferris RL Whiteside TL Ferrone S. Immune escape associated with functional defects in antigen-processing machinery in head and neck cancer. Clin Cancer Res. (2006) 12:3890–5. 10.1158/1078-0432.CCR-05-2750 16818683

[3] Ferris RL Blumenschein G Jr Fayette J Guigay J Colevas D Licitra L. Nivolumab for recurrent squamous-cell carcinoma of the head and neck. N Engl J Med. (2016) 375:1856–67. 10.1056/NEJMoa1602252 27718784 PMC5564292

[4] Cohen EE Harrington KJ Le Tourneau C Dinis J Licitra L Ahn M Pembrolizumab (pembro) vs. standard of care (SOC) for recurrent or metastatic head and neck squamous cell carcinoma (R/M HNSCC): Phase 3 KEYNOTE-040 trial. Ann Oncol. (2017) 28 (suppl.5): v605–49. 10.1093/annonc/mdx440

[5] Uetrecht J. Immune-mediated adverse drug reactions. Chem Res Toxicol. (2009) 22:24–34. 10.1021/tx800389u 19149477

[6] Belkhir R Burel SL Dunogeant L Marabelle A Hollebecque A Besse B. Rheumatoid arthritis and polymyalgia rheumatica occurring after immune checkpoint inhibitor treatment. Ann Rheum Dis. (2017) 76:1747–50. 10.1136/annrheumdis-2017-211216 28600350

[7] Cappelli LC Gutierrez AK Bingham CO III Shah AA. Rheumatic and musculoskeletal immune-related adverse events due to immune checkpoint inhibitors: a systematic review of the literature. Arthritis Care Res. (2016) 69:1751–63. 10.1002/acr.23177 27998041 PMC5478477

[8] Cappelli LC Shah AA Bingham CO III. Immune-related adverse effects of cancer immunotherapy- implications for rheumatology. Rheum Dis Clin North Am. (2017) 43:65–78. 10.1016/j.rdc.2016.09.007 27890174 PMC5127444

[9] Cappelli LC Gutierrez AK Baer AN Albayda J Manno RL Haque U. Inflammatory arthritis and sicca syndrome induced by nivolumab and ipilimumab. Ann Rheum Dis. (2017) 76:43–50. 10.1136/annrheumdis-2016-209595 27307501 PMC5333990

[10] Gauci ML Baroudjian B Laly P Madelaine I Da Meda L Vercellino L. Remitting seronegative symmetrical synovitis with pitting edema (RS3PE) syndrome induced by nivolumab. Semin Arthritis Rheum. (2017) 47:281–7. 10.1016/j.semarthrit.2017.03.003 28438383

[11] Calabrese C Kirchner E Kontzias K Velcheti V Calabrese LH. Rheumatic immune-related adverse events of checkpoint therapy for cancer: case series of a new nosological entity. RMD Open (2017) 3:e000412. 10.1136/rmdopen-2016-000412 28405474 PMC5372131

[12] Chan MM Kefford RF Carlino M Clements A Manolios N. Arthritis and tenosynovitis associated with the anti-PD1 antibody pembrolizumab in metastatic melanoma. J Immunother. (2015) 38:37-9. 10.1097/CJI.0000000000000060 25415286

[13] Baxi S Yang A Gennarelli RL Khan N Wang Z Boyce L. Immune-related adverse events for anti-PD-1 and anti-PD-L1 drugs: systematic review and meta-analysis. BMJ (2018) 360:k793. 10.1136/bmj.k793 29540345 PMC5851471

[14] Sosa A Lopez Cadena E Simon Olive C Karachaliou N Rosell R. Clinical assessment of immune-related adverse events. Ther Adv Med Oncol. (2018) 10:1758835918764628. 10.1177/1758835918764628 29623110 PMC5882039

[15] Zimmer L Goldinger SM Hofmann L Loquai C Ugurel S Thomas I. Neurological, respiratory, musculoskeletal, cardiac and ocular side-effects of anti-PD-1 therapy. Eur J Cancer (2016) 60:210–25. 10.1016/j.ejca.2016.02.024 27084345

[16] Hua C Boussemart L Mateus C Routier E Boutros C Cazenave H. Association of vitiligo with tumor response in patients with metastatic melanoma treated with pembrolizumab. JAMA Dermatol. (2016) 152:45–51. 10.1001/jamadermatol.2015.2707 26501224

